# Good Samaritans in Networks: An Experiment on How Networks Influence Egalitarian Sharing and the Evolution of Inequality

**DOI:** 10.1371/journal.pone.0128777

**Published:** 2015-06-10

**Authors:** Yen-Sheng Chiang

**Affiliations:** Department of Sociology, The Chinese University of Hong Kong, Hong Kong, China; University of Maribor, SLOVENIA

## Abstract

The fact that the more resourceful people are sharing with the poor to mitigate inequality—egalitarian sharing—is well documented in the behavioral science research. How inequality evolves as a result of egalitarian sharing is determined by the structure of “who gives whom”. While most prior experimental research investigates allocation of resources in dyads and groups, the paper extends the research of egalitarian sharing to networks for a more generalized structure of social interaction. An agent-based model is proposed to predict how actors, linked in networks, share their incomes with neighbors. A laboratory experiment with human subjects further shows that income distributions evolve to different states in different network topologies. Inequality is significantly reduced in networks where the very rich and the very poor are connected so that income discrepancy is salient enough to motivate the rich to share their incomes with the poor. The study suggests that social networks make a difference in how egalitarian sharing influences the evolution of inequality.

## Introduction

The proliferation of research in the behavioral sciences of the past decade has provided strong evidence to the human nature of aversion to economic inequality and the propensity to care for the economic disadvantaged. Experimental studies show that from children to adults people share valuable goods with unrelated others [1–7] and take costly action to correct unfair divisions of resources between strangers from which they claim no benefits [8–10]. These studies suggest that facing unequal distributions people are willing to sacrifice their own benefits to help the economic disadvantaged—an action termed egalitarian sharing [1,11–14], although egalitarianism is far from the only guiding principle of human behavior, as people are also found to choose utilitarian options that maximize group welfare over egalitarian divisions of resources [15–16].

Most of the experimental research on egalitarian sharing investigates the division of resources in a dyad, in which a person interacts with an alter, or a complete group, wherein actors interact with one another. Social interaction, however, could take a different form than dyads and groups. In fact, many social activities are carried out in networks—a more generalized structure of social interaction, of which dyads and complete groups are two special cases. Network is not only more representative of how social connections are structured, but is also an important mechanism for the emergence of social behavior, such as cooperation and influence [17–20]. Yet, in the literature little do we know how network structure influences the behavior of egalitarian sharing and how inequality evolves in networks.

Social networks play an important role in the assessment of income inequality. The study of social comparison in social psychology indicates that people tend to select particular referents to compare their well-being with [21–23]. Accordingly, scholars have long proposed a network approach to understanding the choices of referents in social evaluation [24]. Network influences not only how social referents are chosen, but also how kindness and generosity flow. Different forms of social supports, such as food sharing, are provided through social networks [25–26]. Economic aids provided by microcredit finances, for example, are mobilized mainly through interpersonal networks [27–28]. These examples suggest that network influences the extent to which inequality is perceived, as well as how altruistic giving is distributed to the needy.

We present an experimental study to investigate how actors share incomes with neighbors in some stylized networks. The result shows that income distribution evolves differently across networks. People’s behavior of sharing is driven by some factors related to the distributions they are exposed to, but the factors are activated of different extents in different networks, explaining in part why there is a difference across networks in how inequality evolves.

## Egalitarian Sharing in Networks

The scenario (or a game) we depict for studying egalitarian sharing in networks can be described as follows. Consider a group of actors, each of which is given an income and linked to a set of others in the group. The network that governs people’s interaction is fixed. In each round of the game, actors view the income distribution of their network neighbors—those linked to them—and decide whether to give money to neighbors. Actors’ incomes are modified whenever they give or receive money from others. The game continues until no one gives.

Understanding how egalitarian sharing is practiced and in turn how income distribution evolves in the game requires considerations of an array of factors that can be summarized in the following two inquiries: What motivates people to share? And whom would they share with?

When actors are placed in a network, the income distribution of their network neighbors is what they are exposed to. Prior research in the economic behavioral sciences has provided insights into how altruistic sharing is influenced by properties related to the distribution itself and the position that allocators take in the distribution. First, behavioral economists found that aversion to inequality is a propellant of prosocial behavior [12, 29]. Larger income discrepancy is expected to trigger more sharing of income. Second, income status could influence the decision. Psychological research found that social status is associated with altruism. An actor who occupies a higher position in the distribution may be more [30] or less [31] motivated to share his/her income with the poor. Thirdly, how many recipients an actor is exposed to could make a difference. A recent study shows that people’s altruism varies with the number of recipients. A person may feel more motivated to give when there are more recipients available [32].

Network not only influences a person’s motive of giving, it also determines the pool of potential recipients. Research evidence suggests that the probability of receiving donation is a function of economic status: the poorer a person is, the more likely s/he would receive giving from others [3, 33]. On the other hand, altruism can be congestible in the sense that similarly poor people are poised to compete for giving from the same giver [32]. The probability of receiving giving, therefore, is not only determined by the recipient’s income level, but is also contingent on how many other similarly poor people are competing for the giving. Givers, on the other hand, could choose different forms to allocate their giving. For example, they could evenly divide the giving to a set of similarly poor people or could randomly select one of them to concentrate their giving. It remains an empirical question how giving would be allocated.

Furthermore, giving does not necessarily come from the rich to the poor per se. Earlier research evidence has found incidents of reverse redistribution; i.e., donation goes along the opposite direction from the poor to the rich [2]. Despite being rare, reverse redistribution can be caused by different motives. One of the drivers is reciprocity: people express their gratitude for receiving donation from others by giving money in return even though that the recipients may have higher incomes than they do. In addition, reverse redistribution can be attributed to a desire not to be the poorest person: the poor may choose to give to the rich, but not those poorer than they are, out the fear that their giving to the poorer may make them the poorest in the distribution [34].

While prior research provides useful guidance to predicting how egalitarian sharing unfolds for an income distribution, the overall effect would be determined by network topology, which delineates the different (local) income distributions that each actor would face in his neighborhood. Tracking the dynamics of income distribution as a result of egalitarian sharing in networks is extremely difficult by intuitive reasoning. To the challenge, we draw on an agent-based model to derive some theoretical predictions. Details of the model are reported in the online supporting materials (S2 File). As can be found there, while the evolution of income distributions is influenced by a multitude of factors pertaining to individual’s sharing behavior, the effects of these factors vary across network topologies.

## The Experiment

### Experiment Design

#### Income Distribution

**Fig 1:**
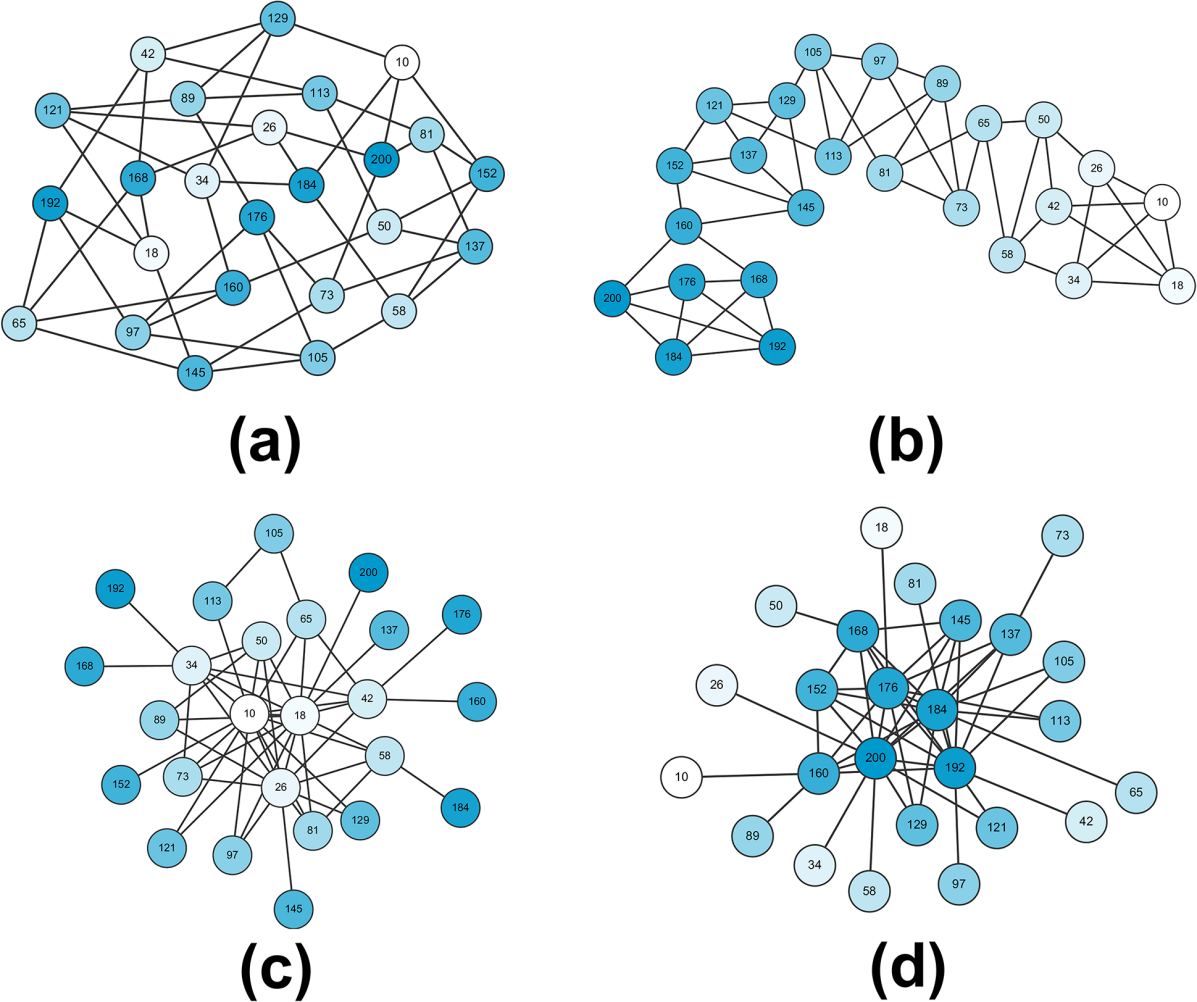
The four network topologies. (a) Lattice_Hetero: actors with discrepant income levels are linked in a lattice, where each node has the same number of ties. (b) Lattice_Homo: actors with similar income levels are linked in a lattice, where each node has the same number of ties. (c) SF_Negative: income levels and nodal degrees are negatively associated in a network where ties are unevenly distributed across nodes. (d) SF_Positive: income levels and nodal degrees are positively associated in a network where ties are unevenly distributed across nodes. Numbers within each node represent income levels. Darker colors refer to higher incomes.

Each actor is given an income in the beginning. Incomes are uniformly distributed (min = 10 and max = 200) over a group of 25 actors, shown by the numbers in each node of the network in Fig 1.

#### Network Topologies

We choose four network topologies that are well studied in network science. For the first two networks, lattices, ties are equally distributed across nodes: each actor is linked to four neighboring others along a circle [35]. For the other two networks, Scale Free Networks (SF), ties are unevenly distributed—while a small number of people are well connected, the remaining are sparsely connected [36]. Owing to their unique structural properties, the two types of networks have proved to influence the emergence of many kinds of social behavior [37–38]. They are selected here for another reason: previous work shows that the number of ties a node has—nodal degree—influences the perception of distributional inequality [39]. Because Lattice and SF networks take opposite positions in the distribution of nodal degree, implementation of the two types of networks allows us to investigate how inequality in the distribution of network ties influences egalitarian sharing.

Within the first network type, lattice, we make a distinction by how incomes are assorted in network. People can be linked with others with little or large difference in incomes—homophily vs. heterophily [40]. In homophilous (heterophilous) networks, an actor’s income would be less (more) different from his neighbors than non-neighbors. The difference between homophily and heterophily is expected to generate different perceptions of local income inequality and mobilize different amounts of giving.

Within the second network type (SF), where ties are unevenly distributed, we make a distinction by how nodal degree and income level are related. Richer people could be more or less linked than the poor in network [41–42]. We simulate the two conditions by relating nodal degree to income level positively and negatively respectively.

Fig 1 presents the four network topologies. Details of the generation of the four networks are provided in the online supporting material (S1 File). The four networks are identical in network density, allowing us to investigate how *structure* rather than the amount of ties influences egalitarian sharing and the evolution of inequality. We also consider a fully saturated network (not shown in the Figure) to represent that everyone is linked to all others in the group. A total of five network topologies are investigated.

### Experimental Procedure

A total of 162 undergraduate students of a public university in southern California of the U.S were recruited to participate in the experiment using monetary payoff as incentive. The experiment was approved by the university IRB (HS#2011–8378). Participants were recruited by a social science experimental laboratory of the university and were allocated to seven sessions. We customized our experiment to accommodate any number of participants that would show up in a session. We worked to recruit 25 participants for each session; however, fewer participants than expected turned out in the last two sessions. For the two sessions with fewer participants, the experiments were run on smaller networks (19 and 18 nodes respectively); except for this difference, every other experiment condition was kept the same as the normal experiment with *n* = 25. The generation of the slightly smaller networks is following the same mechanism detailed in the online supporting materials.

We adopt a within-subject design: in each session, all participants went through five trials, each of which implemented one of the five network topologies (or treatment). The order of the five network trials in a session was randomized. At the end of each session, a network trial was chosen by lottery and participants were paid in proportion to their income levels at the final round of the chosen trial [43].

The experiment was held at a social science laboratory on campus. Participants were seated in individual cubicles and interacted with others through Internets using the pseudo-identities we provided. We customized a web-based experiment program to operate the experiment.

We read out the instruction to participants before the experiment began (the instruction sheet provided in S3 File). In the beginning of an experiment trial, participants were given an income as was specified in Fig 1. Incomes were represented by tokens and participants were told that the tokens were redeemable to money. In each round, the experiment identities of each person’s network neighbors and their current token balances were shown on the screen. If an individual would like to donate token(s) to a network neighbor, s/he could put a number in the box designated for the recipient neighbor. Our program would block illegal inputs, such as symbols, non-integers or negative integers. Shall an illegal input occur, a warning message would pop up and request the subject to input a new donation if s/he wants. The default amount of donation is set to zero so if a person does not input any number, nothing will be donated. The participants were not allowed to give more than they currently had.

Each person has sufficient time (40 seconds) to make a decision of giving in each round. The game moves to the next round when all participants have made their decisions or when the time expires. The game stops under two circumstances: either when no one gives, or the game finishes the 10^th^ round. The former condition is an ideal stopping rule, but to prevent the game from proceeding too long, we imposed a compulsory stopping time at round 10 if the experiment fails to stop by then. The participants were informed of the first stopping rule, but did not know of the compulsory stopping rule set at round 10.

Participants were paid individually at the end of the experiment. The payoff includes a show-up fee (US$7), plus the token balance in the last round of the chosen trial. On average, a participant received $12.25 from the experiment.

### Experiment Result

A total of 35 experiment trials (7 sessions × 5 trials) were run. Four of them encountered unexpected software problems in the middle of the experiment. The failed trials were not included in the analysis.

#### Inter-temporal Distribution of Giving

S7and S8 Figs present the records of giving over time. About half of the participants donated money in the early period of the experiment. The proportion drops to around 20% by round 10. On average, people donated 5.4% of their incomes in the beginning, and the percentage falls to 2.6% by round 10. In 7 of the 31 experiment trials that were successfully run (22%), all participants stopped giving before round 10.

#### End-Round Inequality

**Fig 2:**
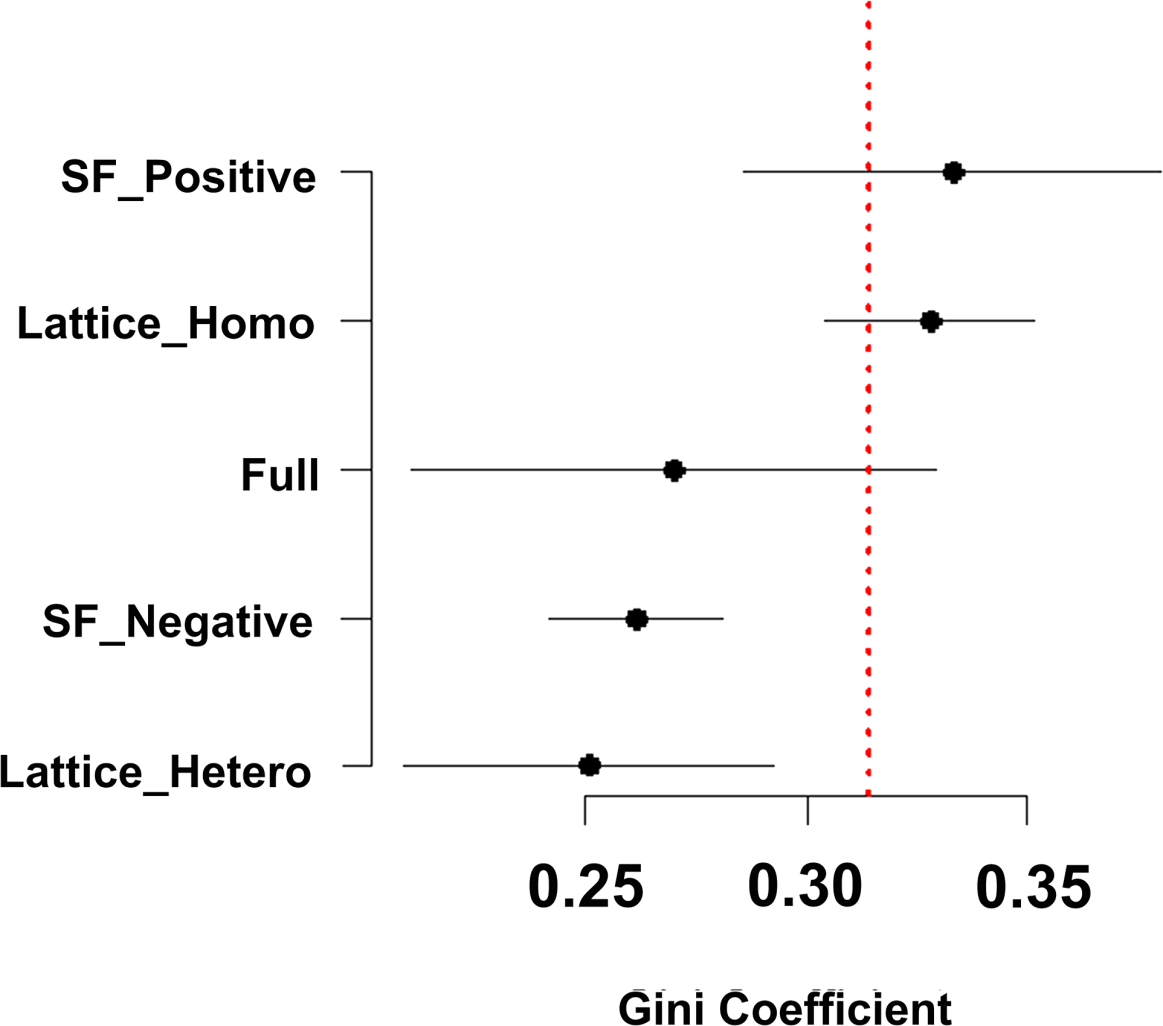
Inequalities of the end-round distributions measured by the Gini coefficient for each network treatment. The segments represent the 95% confidence interval. The vertical dotted line shows the inequality level of the original distribution.

Our primary objective is to compare income distributions in the initial and the final round of the experiment to see whether inequality improves or not. Fig 2 presents the distribution of inequality levels measured by the Gini coefficient for each network treatment. We calculate the Gini coefficient of the end-round distribution for each session. Using session as the unit of analysis, we compare the initial and the end-round Gini coefficients by running the Wilcoxon Signed Rank test (for more details, please see S4 File). The test shows that the Gini coefficient of the end-round distribution is lower than the original income distribution in the Lattice_Hetero and the SF_Negative network treatment (W = 0, p = 0.01 and W = 0, p = 0.03), but not in the other three network treatments (W = 5; p = 0.31 for Full; W = 15; p = 0.44 for Lattice_Homo and W = 14; p = 0.56 for SF_Positive).

The finding shows a difference in the reduction of inequality across the five network treatments. Why is there such a difference? We attempt to seek the answer by looking into subjects’ behavior of sharing in the experiment. As would be shown, the two networks found to experience a significant reduction of inequality actually performed differently from others in triggering actors’ egalitarian sharing in the experiment.

#### Individuals’ Behavior

In reference to the discussion in section 2, here we consider a list of variables that are suspected to trigger subjects’ sharing of incomes: Actor *i’*s income (*X*_*i*,*t*_) and nodal degree (*K*_*i*_); the ranking of actor *i* (*R*_*i*,*t*_) and the inequality level (*L*_*i*,*t*_) of the income distribution in actor *i*’s network neighborhood. The subscript *t* (time) denotes that the variable is endogenous and subject to change in each round.

Actor *i’*s income level at time *t* (*X*_*i*,*t*_) is bound between 0 and the sum of all actors’ incomes. Income ranking (*R*_*i*,*t*_) is the position that actor *i* takes in the sequence, ordered from low to high, of the incomes of actor *i*’s and his network neighbors. We normalize the ranking by dividing it by the length of the sequence so that *R*_*i*,*t*_ would be bound between 0 and 1. Local inequality (*L*_*i*,*t*_) is the Gini coefficient of the income distributions of actor *i* and his neighbors. Nodal degree (*K*_*i*_) is the number of ties linked to actor *i*.

The variables, *X*_*i*,*t*_, *R*_*i*,*t*_ and *L*_*i*,*t*_, represent perception of inequality of different levels [39]: *X*_*i*,*t*_ is actor *i*’s own income; *R*_*i*,*t*_ is a comparison of *i’*s income with others’, and *L*_*i*,*t*_ extends the comparison to all neighbors, which takes into account the income difference among one another in the neighborhood. Egalitarian sharing is possible to be triggered by the three different perspectives to inequality.

Theoretical predictions of how the variables above determine the evolution of incomes in different networks can be found in the online supporting materials, assuming that these factors take effect. Yet, whether these factors significantly influence participants’ decision-making of giving in each round remain an empirical question. To the question, I use a Hurdle regression model to assess the effects of these factors. In the Hurdle regression, the probability and the amount of giving are assessed separately and the latter is estimated only when the former passes a threshold [3, 44]. In our within-subject design, the decisions of giving are not independent so standard errors of the regression coefficients are clustered within subjects in the following analysis.

Tables 1 and 2 shows the Hurdle regression result on participants’ giving in each round. The variables perform differently across networks. Notably, the two networks, Lattice_Hetero and the SF_Negative, differ from other networks in local inequality (*L*): both the coefficients are positive in estimating the probability and the amount of giving, suggesting that high local inequality would prompt a person to give more in the two networks, but not in others. As can be found in the online supporting materials, a positive coefficient of local inequality (*L*_*i*,*t*_) contributes to the mitigation of inequality. It explains in part why inequality can improve more profoundly in the two networks.

**Table 1:** Hurdle Regression Model on Giving Decisions (Probability of Giving).

			Networks		
	Full	Lattice_Hetero	Lattice_Homo	SF_Negative	SF_Positive
Income Level (*X*)	0.006	-0.01**	0.002	-0.004	0.005
Income Ranking (*R*)	-2.27*	1.28*	-0.68	0.80	1.45*
Local Inequality (*L*)	6.44	4.28***	1.36	4.64***	1.26
Nodal Degree (*K*)	-0.08	N/A	N/A	0.09*	-0.0006

*Note*: *** p<0.*001*

** p<0.*01*

* p<0.*05*.

**Table 2:** Hurdle Regression Model on Giving Decisions (Amount of Giving).

			Networks		
	Full	Lattice_Hetero	Lattice_Homo	SF_Negative	SF_Positive
Income Level (*X*)	0.002	-0.0002	0.0003	-0.0003	-0.007
Income Ranking (*R*)	0.21	-0.06	-0.53	-0.60	-0.09
Local Inequality (*L*)	-1.29	2.93**	1.01	4.61***	-2.05
Nodal Degree (*K*)	0.08	N/A	N/A	-0.08**	0.10

*Note*: *** p<0.*001*

** p<0.*01*

* p<0.*05*.

But why do the two networks motivate people to respond to local inequality more vividly than other networks? Part of the answer lies in the inherent local inequality of the two networks. As can be seen in Fig 1, the two networks link together very rich and very poor actors and thus create profound income discrepancies in actors’ local neighborhoods. We suspect that egalitarian sharing is triggered when (local) inequality is large enough, such as in the two networks mentioned above.

Nodal degree (*K*) has a positive and a negative effect respectively on the probability and the amount of giving in the SF_Negative network. Note that in this network the poor are more linked than the rich. The fact that the poor are more likely to give in this network suggests incidence of reverse redistribution. As would be discussed later, reverse redistribution may be motivated by reciprocity: as the poor have received giving from multiple sources in this particular network, they may feel obligated to return the favors even just little. Although S5 Fig indicates that a positive coefficient of the variable *K*_*i*_ helps to improve inequality, the magnitude of the coefficient is so trivial that it does not cause a large impact in the experiment.

Although we found a significant effect of income ranking (*R*) on giving in some of the networks, judged by the sign and the magnitude of it and in reference to S3 Fig, it causes only a minor impact on the reduction of inequality.

How would people allocate their giving to the neighbors? We fit the participants’ donation decisions to the Beta distribution to get some answers. Manipulated by two parameters (denoted by *β*_*1*_ and *β*_*2*_), the Beta distribution encompasses a wide range of distributional patterns, such as right- or left-skewed, uniform and bi-modal distributions. An empirical assessment of the participants’ allocation of giving would help us understand how people select recipients of their donations.

We fit the data of the recipients of giving to the Beta distribution. The best-fit values of the parameter *β*_*1*_ and *β*_*2*_, reported in Table 3, indicate that the distributions are left-skewed (shown in S1 Fig). The pattern suggests that people tend to allocate a high proportion of giving to the relatively poor in their local neighborhood, except for the SF-Positive network, for which the distribution is more bi-modal.

**Table 3:** Fitted Parameters of the Beta Distribution.

			Networks		
	Full	Lattice_Hetero	Lattice_Homo	SF_Negative	SF_Positive
*β_1_*	0.21	1.27	0.97	1.06	0.32
*β_2_*	1.03	1.72	1.72	1.65	0.21

Note, however, the fact that a person is allocating his giving to a low income-ranking recipient in networks does not necessarily mean that inequality would be improved equally effectively. The effect would depend on the gradient of income discrepancy. Consider, for example, the contrast between the Lattice_Hetero and the Lattice_Homo network. Despite sharing a similar left-skewed distribution in the choices of recipients of giving, inequality is improved in the former, but not in the latter network, because donation is transferred from the rich to the poor in the former network with a steep income gradient, while in the latter giving is exchanged between people of similar income levels (see S9 Fig).

The choices of the recipients of egalitarian giving also explain why the Full network fails to reduce inequality as profoundly as we expect. In the Full network, an actor is linked to everyone else so he has many choices to share income with. Although the fitted Beta distribution suggests that participants are allocating their giving to the very poor, a closer look into the data indicates that giving in the Full network treatment is still less concentrative than the two leading networks—the Lattice_Hetero and the SF_Negative network. We found that the number of persons who had received a giving was greater, but the average amount of money received was lower in the Full network than the other two networks. It suggests that giving was allocated more evenly to neighbors in the Full network than the Lattice_Hetero and the SF_Negative network. When giving is not generous, such as in our experiment, a more concentrative allocation of the giving to the poor, demonstrated by the Lattice_Hetero and the SF_Negative network, would work better in improving inequality.

#### Reverse Redistribution

In addition to the kind of giving we would expect from the rich to the poor, in the experiment we also found incidents of reverse redistribution (11.67%). As discussed in section 2, reverse redistribution could be triggered by reciprocity. Indeed, the experiment result shows that a person who received more in the previous round tend to have higher amounts of reverse donations in the present round (Hurdle regression, *p* = 0.009 for the probability and *p*<0.001 for the amount of reverse donation). Note that in our experiment participants only knew how much as a total they received in the previous round, but did not know exactly who gave them. Therefore, direct reciprocity to the original givers is impossible, but indirect reciprocity in the form of generalized exchange—returning favors to a third party different from the original donor—is possible to occur in the experiment [45–46] (but also see [47] for opposite evidence).

## Concluding Remarks

The paper presents a laboratory experiment to investigate how people share their incomes to pursue a more equitable distribution in networks. The study extends the convention of studying egalitarian sharing in dyads and groups to the network frontier, motivated by the premise that network is not only a generalized structure of social interaction, but is also an important mechanism driving the emergence of social complexity.

We developed a number of behavioral rules in the model in reference to past research on the behavior of egalitarian sharing. We implemented the rules in the model and found a difference across networks in how inequality evolves. Tested with human subjects, the laboratory experiment confirms that network influences the mitigation of inequality. Some networks perform better than others in improving inequality as they activate more profoundly some of the behavior rules found beneficial for the improvement of inequality in the model. It is noteworthy that there were still a few people donating money before the experiment was terminated in many sessions, implying that inequality would have been mitigated even further had the experiment continued. In fact, the experiments that had higher percentages of people not ceasing from giving at the end were the network treatments found effective in improving inequality, so it suggests that the superiority of these network topologies in improving inequality would have been more profound had we prolonged the experiment time.

The two networks found to improve inequality, the Lattice_Hetero and the SF-Negative networks, suggest that egalitarian sharing can improve inequality in the following ways. First, the poor are immensely linked to the rich so that poor people can receive giving from multiple sources—in the case of the SF-Negative network. Second, the poor are not highly linked, but they are properly segregated from one another so as not to compete for giving from the same donors—in the case of the Lattice_Hetero network. In combination, the findings suggest while it is important to see whom the poor are linked to, it is equally important whom the rich are linked to as well, when it comes to how egalitarian sharing influences inequality improvement in networks.

The five networks manipulated in the paper are selected from the popularly studied network topologies in the physical and social science literature. They serve as examples to show how network topology influences egalitarian sharing and the evolution of inequality. A number of possible directions can be considered to extend the study. On the one hand, future studies can implement the experiment in real social networks. The emergence of social media makes it easier than before to conduct a real-time experiment on egalitarian income redistribution on a larger scale in people’s daily-life social networks. On the other hand, as it is impossible to exhaust all network topologies, a more parsimonious approach is to study how the underlying structural properties of these networks influence individuals’ sharing behavior and the evolution of inequality. As far the model is concerned, one can also consider the cost in addition to altruistic giving so that when one helps the poor, the cost does not only include the giving itself, but also some cost incurred in the transition, such as the management fee paid to charity organizations [48]. Consideration of the transaction cost is expected to influence actors’ behavior of sharing as well as the consequence of how networks influence the mitigation of inequality.

## Supporting Information

S1 Fig Density functions of the Beta distribution. The horizontal axis marks an interval between 0 and 1 and the vertical axis is the density of the distribution. Vectors in the legend of panel (a) show the parameter values of *β*_*1*_ (left) and *β*_*2*_ (right).
(TIF)

S2 Fig The average inequality level (Gini coefficient) of the end-round distribution in the simulation tested against *C*_*X*_ (light coral color) and *C*_*X’*_ (light steel blue color). The shaded areas mark one standard error above and below the means. The horizontal dotted line shows the inequality level of the original distribution.
(TIF)

S3 Fig The average inequality level (Gini coefficient) of the end-round distribution in the simulation tested against *C*_*R*_ (light coral color) and *C*_*R’*_ (light steel blue color). The shaded areas mark one standard error above and below the means. The horizontal dotted line shows the inequality level of the original distribution.
(TIF)

S4 Fig The average inequality level (Gini coefficient) of the end-round distribution in the simulation tested against *C*_*L*_ (light coral color) and *C*_*L’*_ (light steel blue color). The shaded areas mark one standard error above and below the means. The horizontal dotted line shows the inequality level of the original distribution.
(TIF)

S5 Fig The average inequality level (Gini coefficient) of the end-round distribution in the simulation tested against *C*_*K*_ (light coral color) and *C*_*K’*_ (light steel blue color). The shaded areas mark one standard error above and below the means. The horizontal dotted line shows the inequality level of the original distribution.
(TIF)

S6 Fig The average inequality level (Gini coefficient) of the end-round distribution in the simulation tested against *β*_*1*_ (light coral color) and *β*_*2*_ (light steel blue color). The shaded areas mark one standard error above and below the means. The horizontal dotted line shows the inequality level of the original distribution.
(TIF)

S7 Fig The proportion of participants that had donated in each round of the experiment. The values represent the mean proportions.
(TIF)

S8 Fig The proportion of an individual’s income given to others over the experiment. The Figure plots the mean proportions in each round of the experiment.
(TIF)

S9 Fig The distributions of donations from donors to recipients in the experiment marked by initial income levels. The x-axis (width) represents a donor’s initial income levels and the y-axis (depth) shows a recipient’s initial income levels. The accumulated donations delivered from the donor to the recipient are marked on the z-axis (height). Panel (a) shows the Lattice_Hetero network and (b) the Lattice_Homo network.
(TIF)

S1 File Generation of the Network Topologies. (DOCX)

S2 File The Agent-Based Model. (DOCX)

S3 File Experiment Instruction. (DOCX)

S4 File Statistical Analysis of the Experiment Results. (DOCX)

S1 Table Parameter Values Tested in the Simulation. (TXT)
