# Supplementary figures and images for: Good Samaritans in Networks: An Experiment on How Networks Influence Egalitarian Sharing and the Evolution of Inequality
Source: PLoS One. 2015 Jun 10;10(6):e0128777. doi: 10.1371/journal.pone.0128777 (PMC4465669; doi:10.1371/journal.pone.0128777)

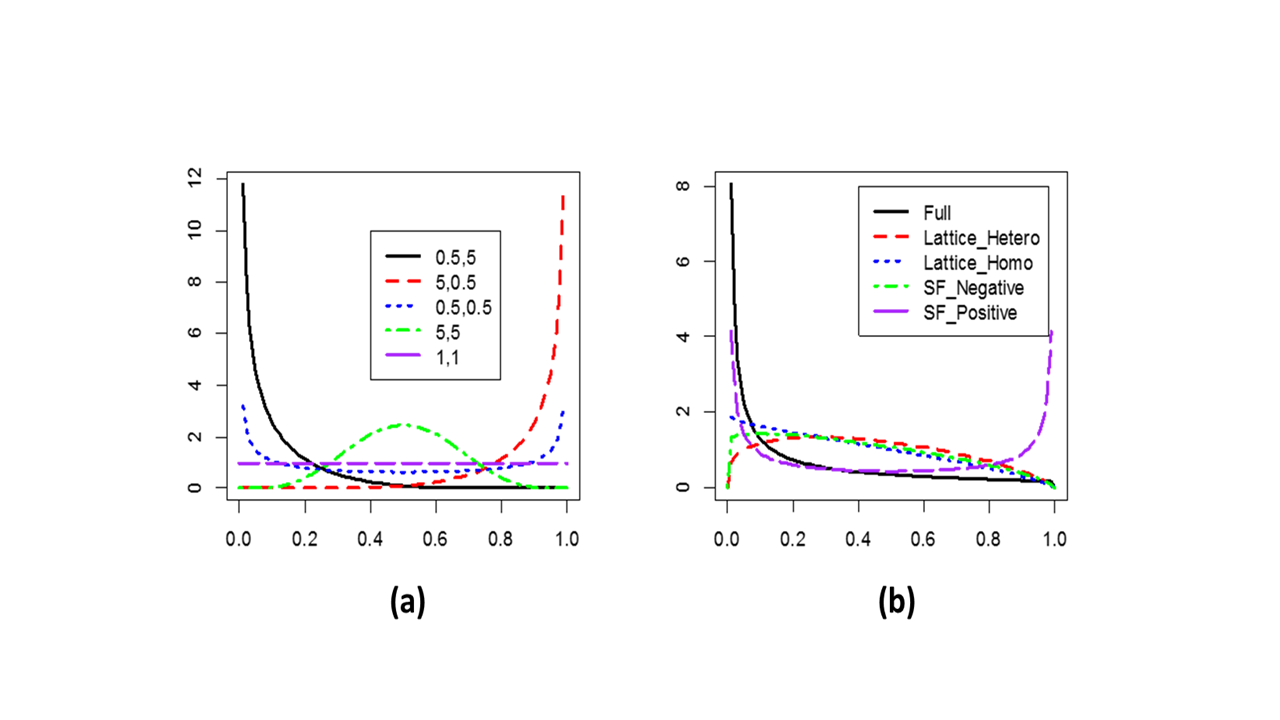

Supplement: S1 Fig — The horizontal axis marks an interval between 0 and 1 and the vertical axis is the density of the distribution. Vectors in the legend of panel (a) show the parameter values of β 1 (left) and β 2 (right). (TIF) [file pone.0128777.s001.tif]

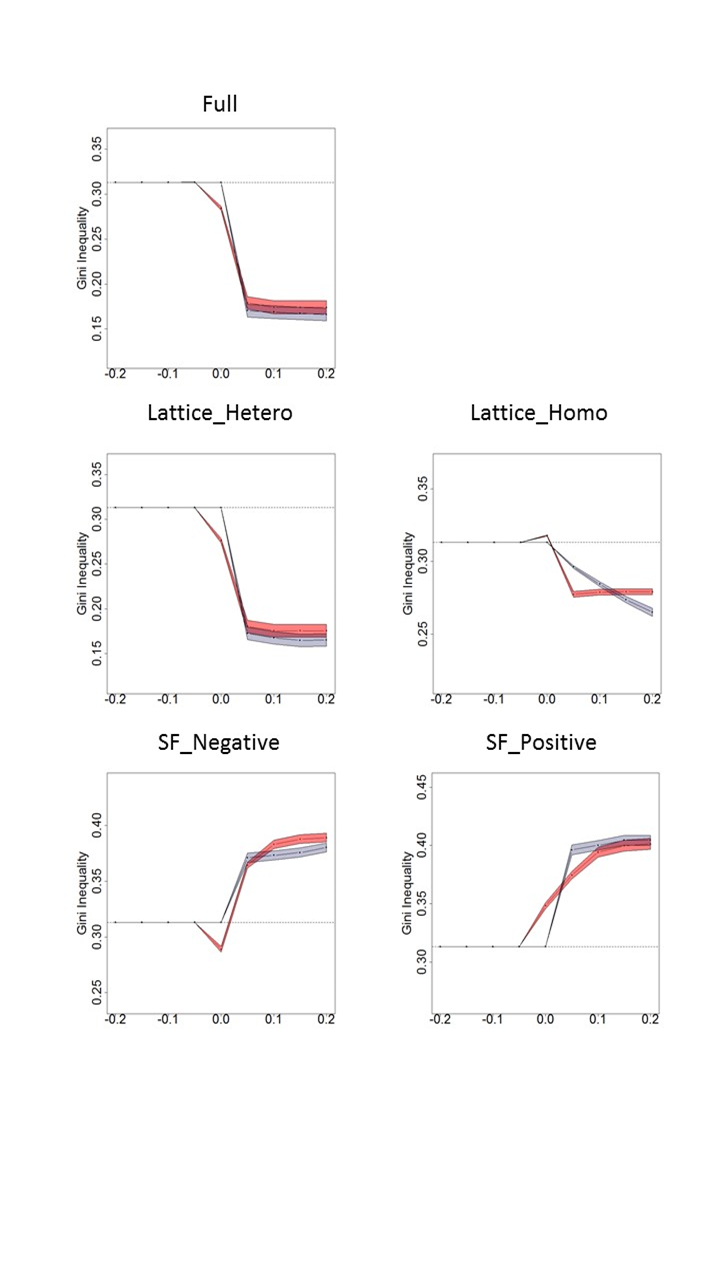

Supplement: S2 Fig — The shaded areas mark one standard error above and below the means. The horizontal dotted line shows the inequality level of the original distribution. (TIF) [file pone.0128777.s002.tif]

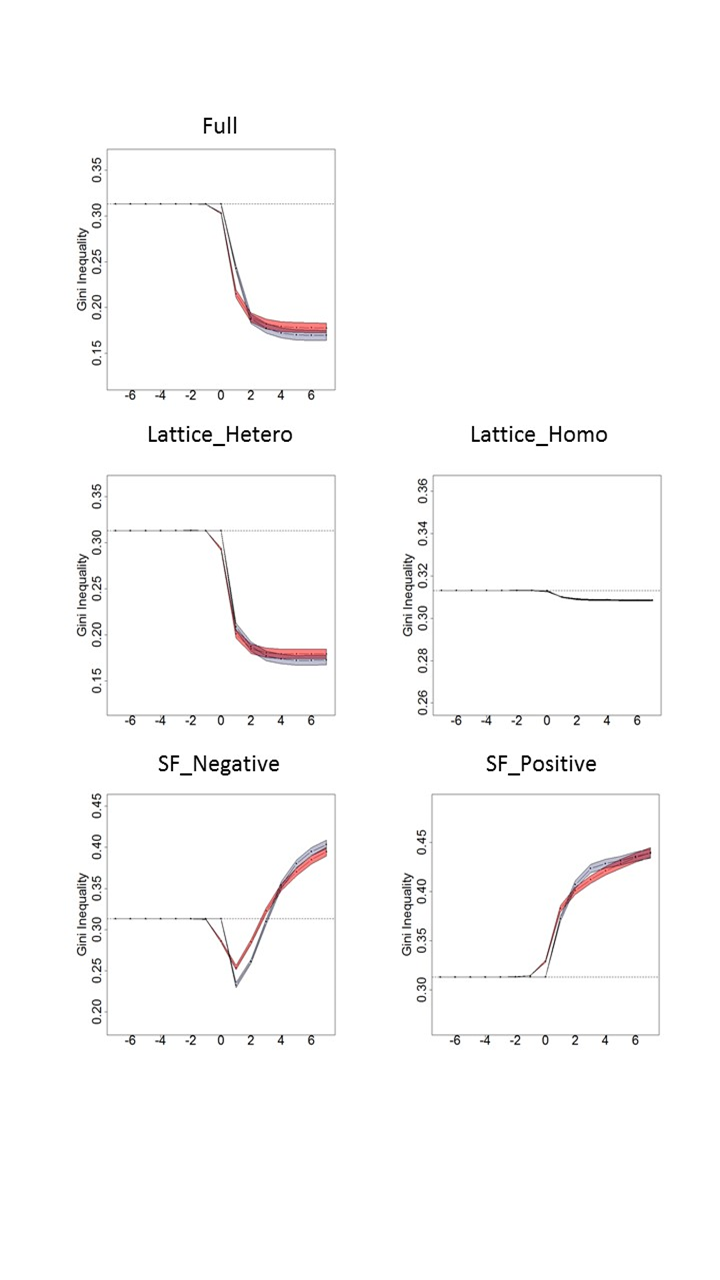

Supplement: S3 Fig — The shaded areas mark one standard error above and below the means. The horizontal dotted line shows the inequality level of the original distribution. (TIF) [file pone.0128777.s003.tif]

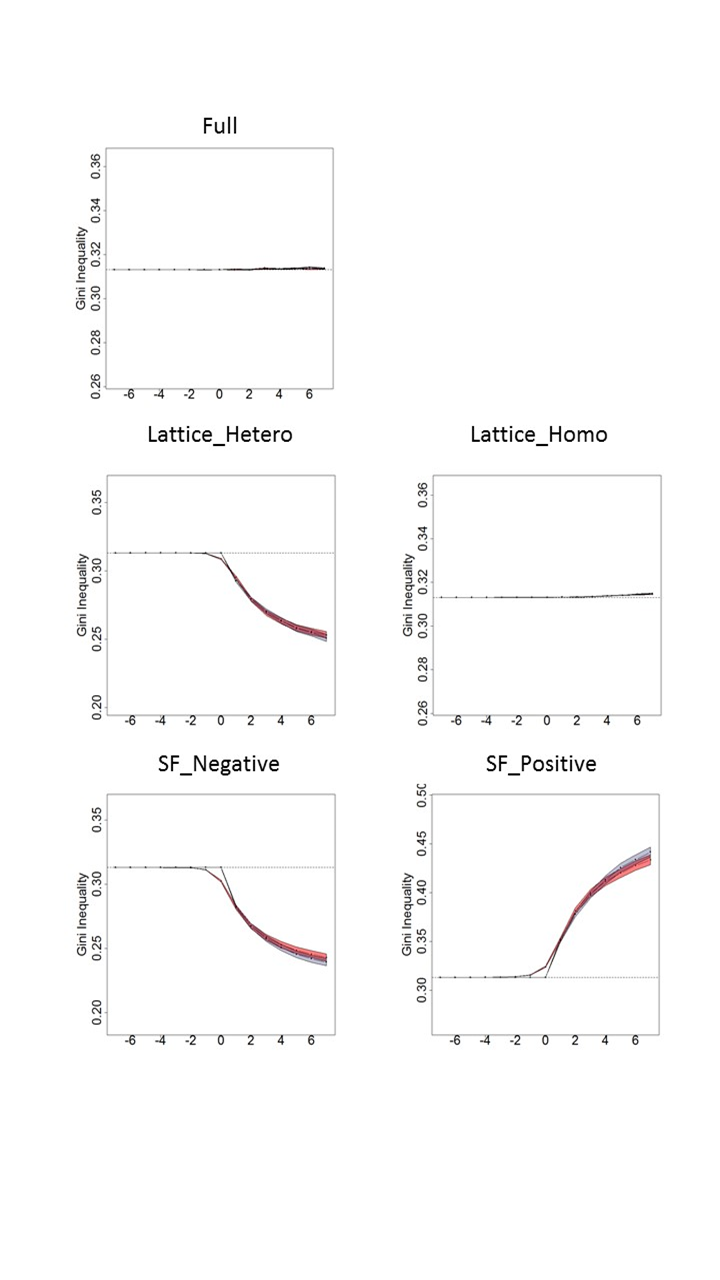

Supplement: S4 Fig — The shaded areas mark one standard error above and below the means. The horizontal dotted line shows the inequality level of the original distribution. (TIF) [file pone.0128777.s004.tif]

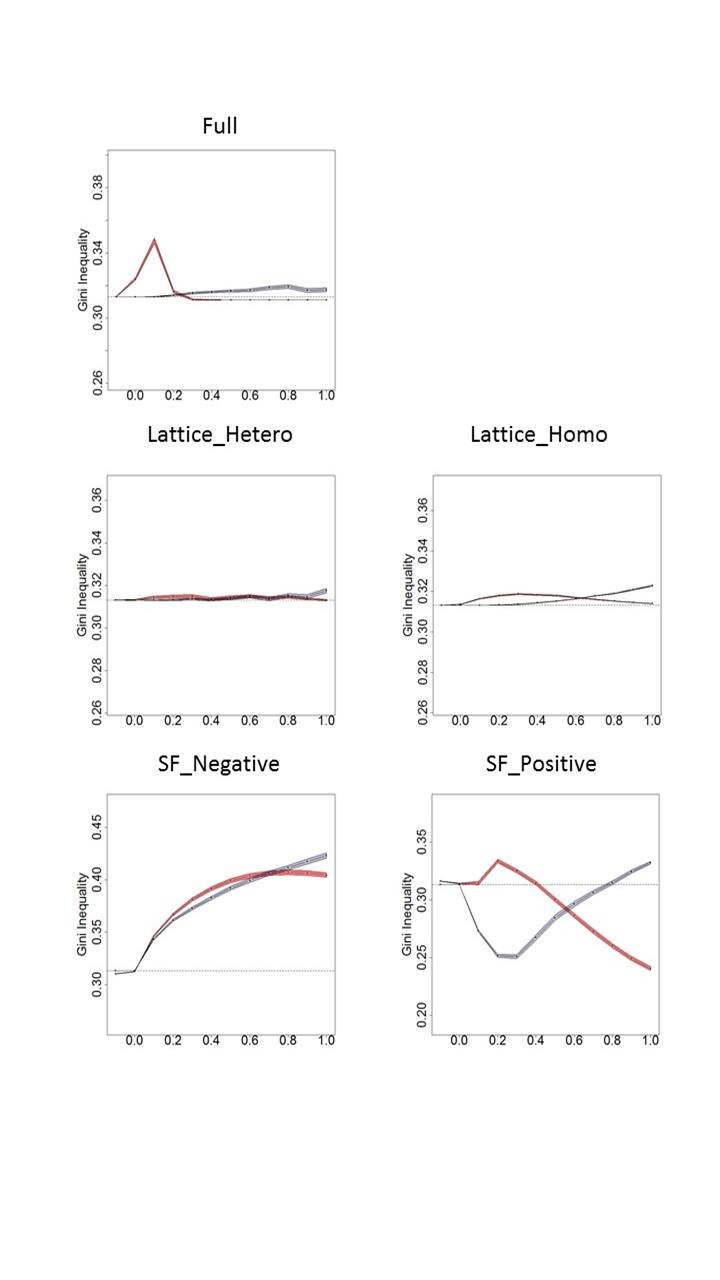

Supplement: S5 Fig — The shaded areas mark one standard error above and below the means. The horizontal dotted line shows the inequality level of the original distribution. (TIF) [file pone.0128777.s005.tif]

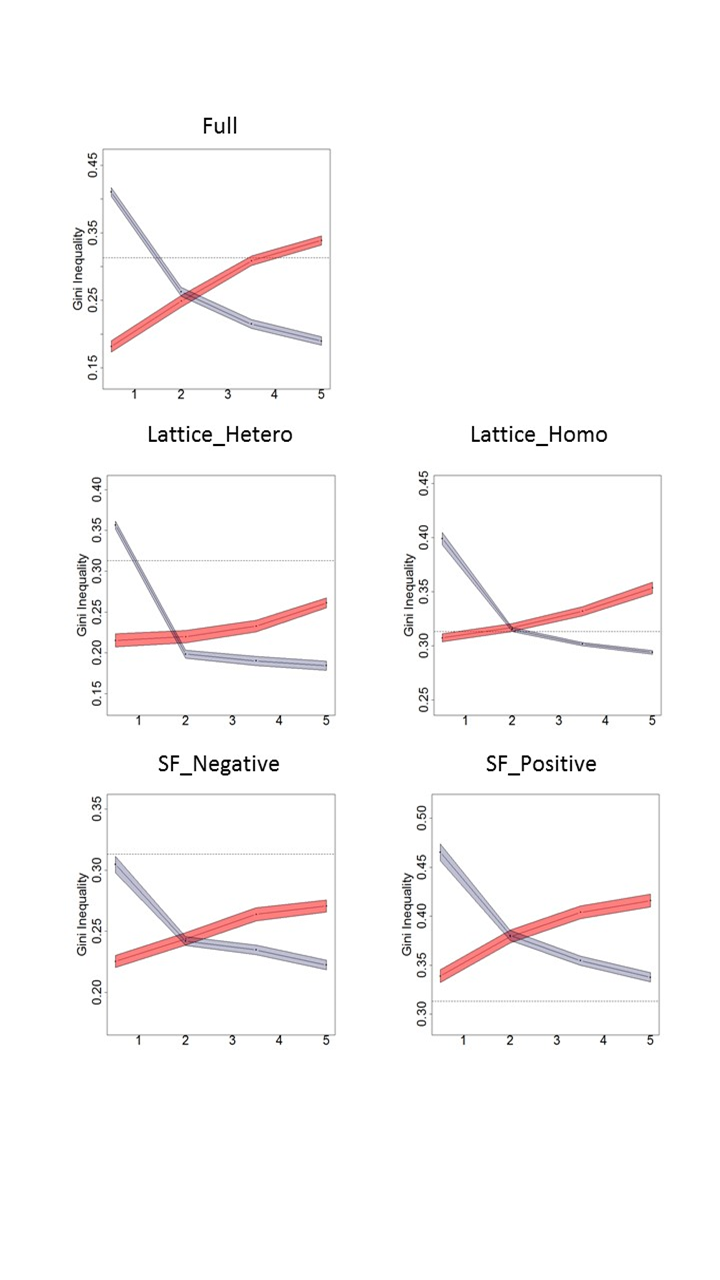

Supplement: S6 Fig — The shaded areas mark one standard error above and below the means. The horizontal dotted line shows the inequality level of the original distribution. (TIF) [file pone.0128777.s006.tif]

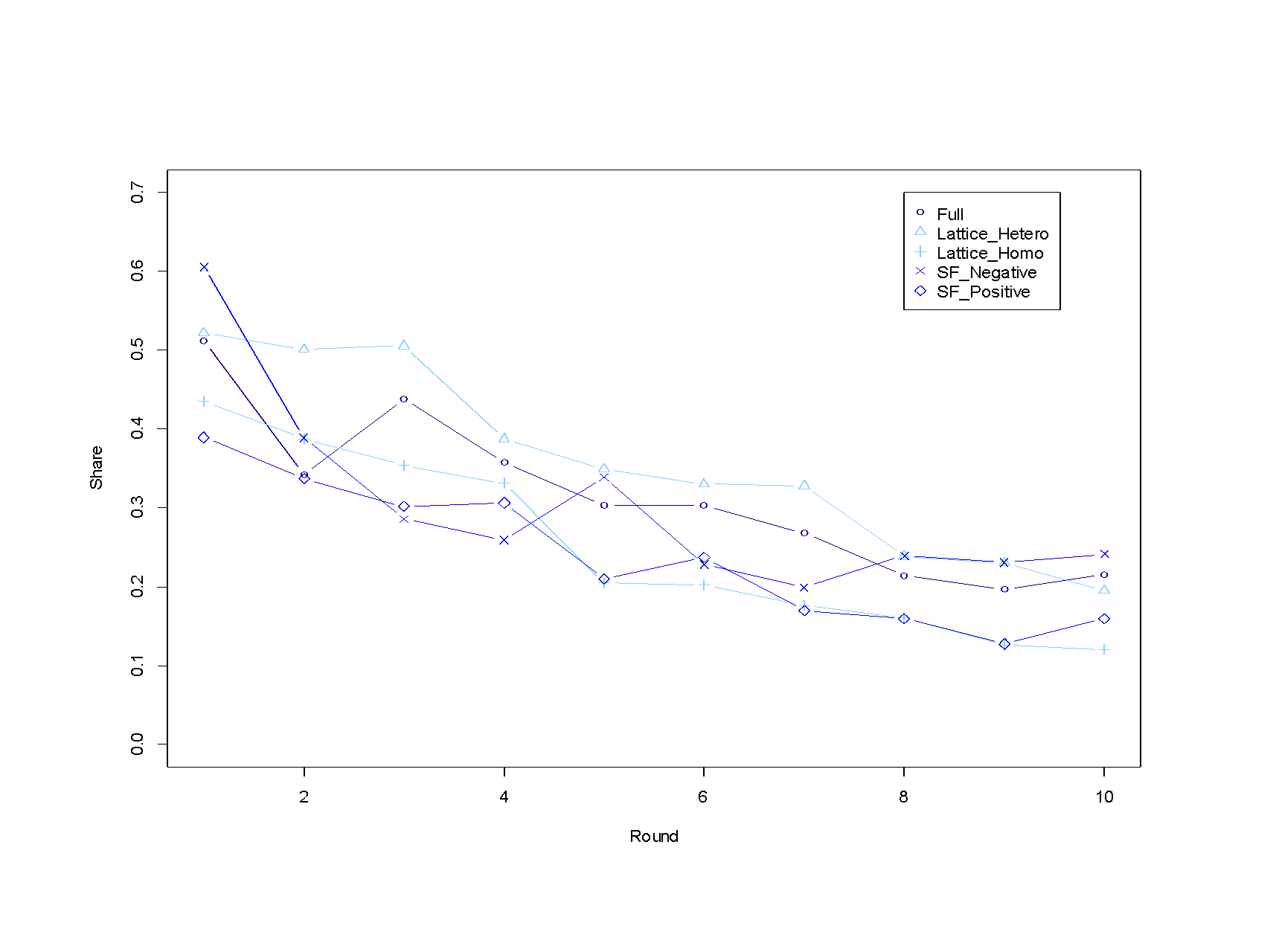

Supplement: S7 Fig — The values represent the mean proportions. (TIF) [file pone.0128777.s007.tif]

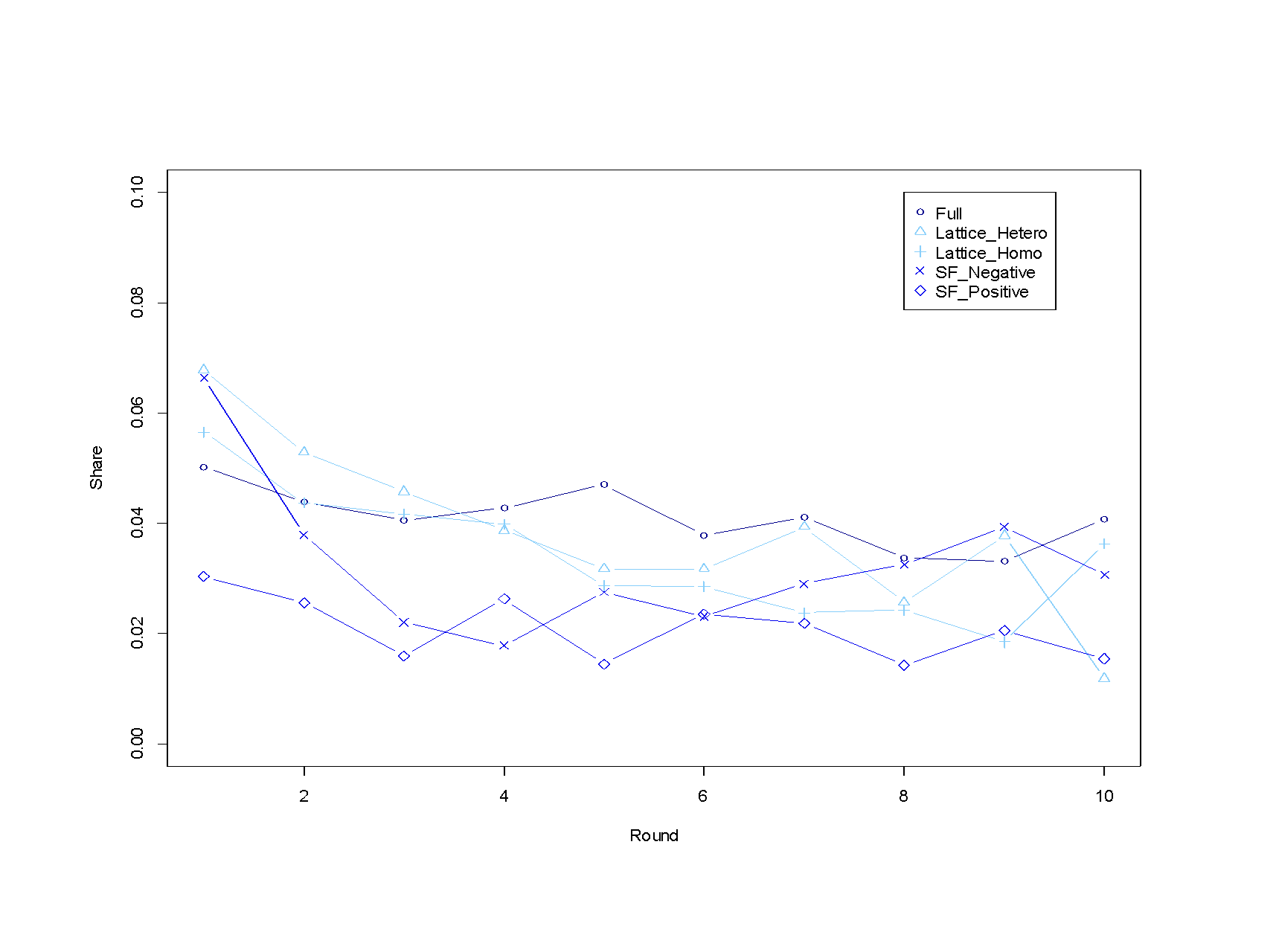

Supplement: S8 Fig — The Figure plots the mean proportions in each round of the experiment. (TIF) [file pone.0128777.s008.tif]

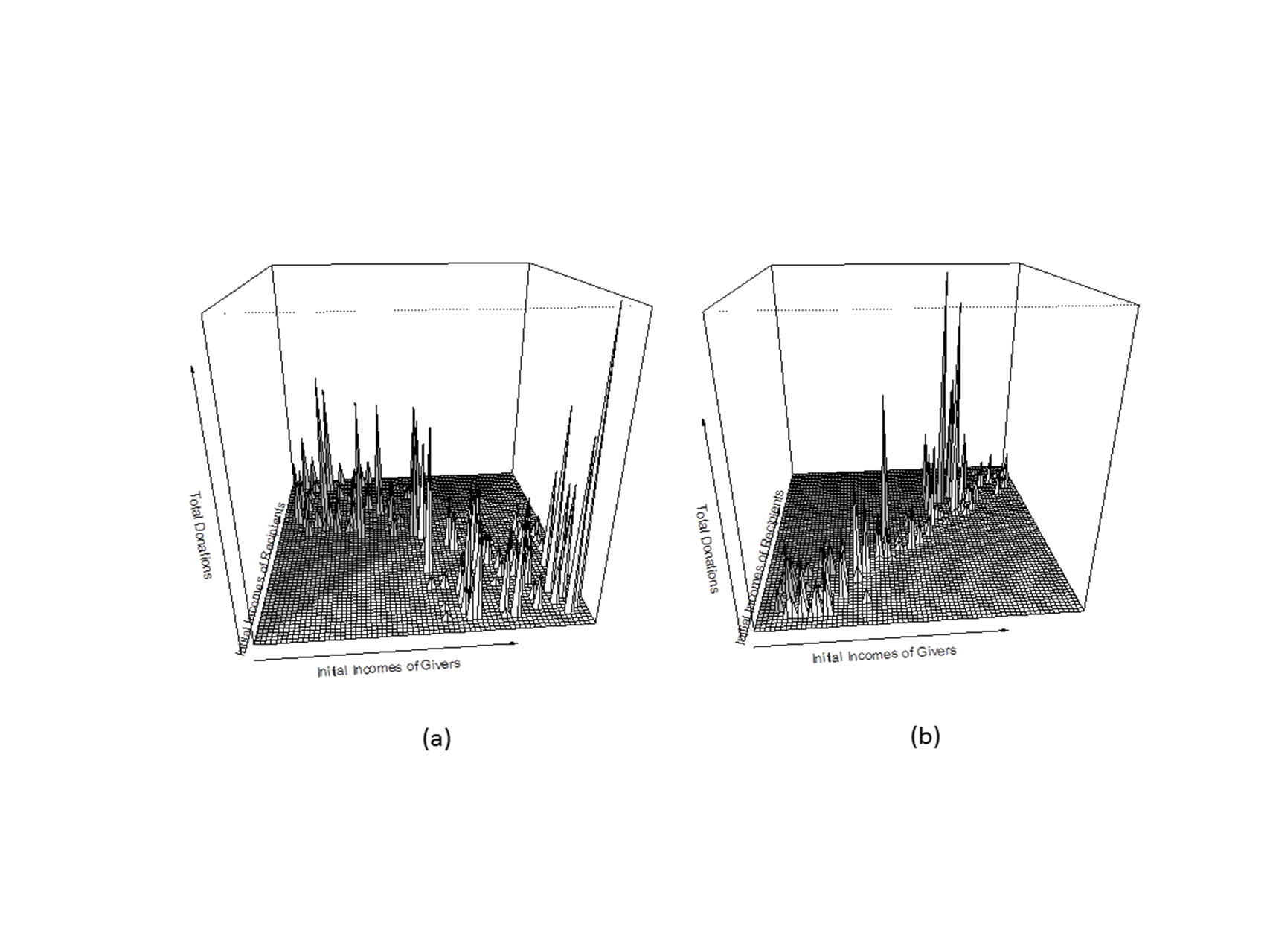

Supplement: S9 Fig — The x-axis (width) represents a donor’s initial income levels and the y-axis (depth) shows a recipient’s initial income levels. The accumulated donations delivered from the donor to the recipient are marked on the z-axis (height). Panel (a) shows the Lattice_Hetero network and (b) the Lattice_Homo network. (TIF) [file pone.0128777.s009.tif]
