# Supplementary material for: Good Samaritans in Networks: An Experiment on How Networks Influence Egalitarian Sharing and the Evolution of Inequality
Source: PLoS One. 2015 Jun 10;10(6):e0128777. doi: 10.1371/journal.pone.0128777 (PMC4465669; doi:10.1371/journal.pone.0128777)
Supplement: S1 File — (DOCX) [file pone.0128777.s010.docx]

**S1. Generation of the Network Topologies (Fig. 1)**

To generate the first two network treatments, where ties are equally distributed, we prepare lattice structures, wherein nodal degree—the number of ties per node—equals to four and network size is set to 25 nodes.

We manipulate the assortment of incomes on network—homophily or heterophily, using the Exponential Random Graph Model (ERGM)—a statistical model developed to assess the structure of empirical networks or simulate particular networks of interest (Robins et al., 2007; Handcock et al., 2008). ERGM accepts a wide range of specification of network formation mechanisms. The specification of network formation is analogous to a logistic regression model, with the dependent variable being the likelihood of a network being formed, independent variables as the mechanisms of network formation and the regression coefficients representing the magnitude of each mechanism. In our case, we consider only one specification in the formation of networks—the discrepancy in income levels between a pair of nodes. This mechanism postulates that, controlled for network density, the probability that a tie exists between two nodes is determined by the difference in incomes between them. If the coefficient is negative (positive), nodes with similar (discrepant) income levels are more likely to be linked. The specification can be formally expressed by the following equation:

$\frac{Prob(e_{ij}=1)}{Prob(e_{ij}=0)}=e^{h d_{ij}}$ (S1)

where *e_ij_*=1 (0) means that node *i* and *j* are (un)linked; *d_ij_* is the absolute difference in income level between node *i* and *j*, and *h* is the regression coefficient. We set the coefficient *h* to -2 and 2 to generate homophilous and heterophilous assortment respectively. For more details on simulating networks using ERGM, readers can refer to Morris et al. (2008).

To generate the second two network treatments, where ties are unevenly distributed, we use algorithm of rewiring ties adapted from the Small-World model (Watts and Strogatz, 1998). Starting with a lattice, we go over each node and rewire each tie, with a certain probability (the first parameter), to a certain target node based on its current nodal degree (the second parameter). Tuning the two parameters to high values, a lattice network would be restructured to the extent that a small number of nodes are highly linked while the remaining ones are poorly linked. This kind of network is also termed “Scale-Free” networks investigated in the physical sciences. For more details of the algorithm outlined here, readers can refer to an example provided in Chiang (2013). Note that because ties are rewired only, the Scale-Free networks generated here do not gain or lose ties from the original lattice networks.

Once the Scale-Free networks are formed, we match individuals’ income levels with nodal degrees in a positive or negative manner: In the first condition, we assign individuals with the highest income to the highest linked node, the second-highest income person to the second highest linked node, so on and so forth. In the other condition, we follow the same procedure in the opposite way. In so doing, we generate two network treatments where ties are unevenly distributed, in one of which richer people are more lined while in the other poor people are more linked.
