# Supplementary material for: Good Samaritans in Networks: An Experiment on How Networks Influence Egalitarian Sharing and the Evolution of Inequality
Source: PLoS One. 2015 Jun 10;10(6):e0128777. doi: 10.1371/journal.pone.0128777 (PMC4465669; doi:10.1371/journal.pone.0128777)
Supplement: S2 File — (DOCX) [file pone.0128777.s011.docx]

**S2. The Agent-Based Model**

**S2.1 Behavior Rules**

We consider a two-step model to address individuals’ behavior of sharing. The model treats the probability and the magnitude of giving as sequent decision modes—analogous to the Hurdle regression model used to analyze the experiment data (Erkal et al., 2011; Gurnu, 1998).

**S2.2 Probability of Giving**

The probability of actor *i*’s giving is treated as a logistic function that takes the following variables: actor *i’*s income level (*X_i,t_*) and income ranking (*R_i,t_*); the inequality level of actor *i’*s network neighborhood (*L_i,t_*), and actor *i’*s nodal degree (*K_i_*). The subscript *t* (time) denotes that the variable is endogenous and subject to change in each round.

$P_{i}=\left(\frac{e^{C_{X}X_{i,t}+C_{R}R_{i,t}+C_{L}L_{i,t}+C_{K}K_{i}}}{1+e^{C_{X}X_{i,t}+C_{R}R_{i,t}+C_{L}L_{i,t}+C_{K}K_{i}}}\right)^{\gamma}$ (S2)

Actor *i’*s income level at time *t* (*X_i,t_*) is bound between 0 and the sum of all actors’ incomes. Income ranking (*R_i,t_*) is the position that actor *i* takes in the sequence, ordered from low to high, of the incomes of actor *i*’s and his network neighbors. We normalize the ranking by dividing it by the length of the sequence so that *R_i,t_* would be bound between 0 and 1. Local inequality (*L_i,t_*) is the Gini coefficient of the income distributions of actor *i* and his neighbors. Nodal degree (*K_i_*) is the number of ties linked to actor *i*.

The parameters (or coefficients), *C_X_*, *C_R_*, *C_L_*, and *C_K_*, control the effects of each of the variables above. A positive value of the parameter suggests that an actor with a higher value in the variable is more likely to give. For example, *C_L_* >0 means that the higher local inequality an actor experiences, the more likely he would share his income.

Finally, the parameter *γ* governs the transformation of the logistic function to the probability of giving. It does not change the nature of the relationship of the parameters with *P_i_*; it merely measures the extent to which the probability of giving is determined by the parameters.

**S2.3 Magnitude of Giving**

The same variables discussed above are used to model the magnitude of giving. The variables, however, are controlled by an independent set of parameters (denoted by the “prime” sign in the equation below). When an actor decides to give, the amount of giving is determined by the following equation:

**$M_{i}=\max\left(\min\left(C_{X'}X_{i,t}+C_{R'}R_{i,t}+C_{L'}L_{i,t}+C_{K'}K_{i}, X_{i,t}\right), 0\right)$** (S3)

wherein max( , ) and min( , ) represents respectively taking the larger and the smaller value between two inputs. The function of min( , ) is used to ensure that actors’ giving does not exceed what they own—no matter how generous they are. On the contrary, max( , ) works to ensure that actors would not give a negative amount—no matter how unwilling they are to give.

**S2.4 Choices of Recipients of Giving**

In each round, for each actor their neighbors’ current incomes are sorted from low to high. Each neighbor *j* has a ranking position (*R_j,t_*) in the sequence. We aim to find a function that would map neighbors’ income ranking to the proportion of giving they would receive from the actor. We found the Beta distribution a suitable candidate to fulfill the goal. Examples of the Beta distributions are provided in Fig. S1a below.

**S2.5 Simulation Setting**

We test how the parameters influence egalitarian sharing and the evolution of inequality in different networks. As the behavior rules addressed here involve 11 parameters, to reduce computational burden, while we manipulate a pair of parameters pertaining to a variable (one for *P_i_* and the other for *M_i_*), we set other parameters that influence the decision of giving to zero and let *β_1_* =1 and *β_2_* =1 to represent a uniform distribution or an unbiased choice of recipients of giving.^[[1]](#footnote-1)^ And *γ*=3 is fixed in all of the tests.^[[2]](#footnote-2)^ The parameter values tested in the simulation are summarized in Table S1.

**S1 Table** Parameter Values Tested in the Simulation

| *C_X_* and *C_X’_* | {-0.20, -0.15,...,0,…, 0.15, 0.20} |
| --- | --- |
| *C_R_* and *C_R’_* | {-7, -6,..., 0,..., 6, 7} |
| *C_L_* and *C_L’_,* | {-7, -6,..., 0,..., 6, 7} |
| *C_K_* and *C_K’_* | {-0.10, -0.08,..,0,.., 0.08, 0.10} |
| *β_1_* and *β_2_* | {0.5, 2, 3.5, 5} |

To recapitulate the process of the game, in the beginning each actor is given an income and placed at a node in one of the five network topologies as illustrated in Fig. 1. In each round, actors decide whether to share their money with neighbors. The probability of giving is determined by equation S2. When actors decide to give, the amount of giving is determined by equation S3. How the giving is allocated proportionally to the neighbors is governed by the Beta distribution (controlled by two parameters, *β_1_* and *β_2_*). Actors’ decision-making is synchronous in the following sense: actors make decisions in reference to the income distribution of the previous round and actors’ incomes are updated together when each person is done with the decision-making of giving in the current round. The game proceeds until no one gives money. Note, however, under certain circumstances the game would not stop endogenously, but would fluctuate between different states.^[[3]](#footnote-3)^ To solve the problem, we impose a compulsory stopping time at round 100 if the game does not terminate by then.

**S2.6 Simulation Results**

We run 50 random simulations for each pair of the parameters shown in Table S1. In each simulation, we report the inequality level, measured by the Gini coefficient, of the income distribution of the final round. Fig. S2-S6 below show the end-round inequality levels against the parameters for each network topology.

1. The exception goes when we test *β_1_* and *β_2_*. Manipulating *β_1_* and *β_2_* while setting all other parameters to 0 leads to no giving at all. To solve the problem, we tune some of the parameters to a modest positive value (*C_X’_* = 0.05, *C_R’_* = 1, *C_L’_* = 1, and *C_K’_* = 0.02) to see how*β_1_* and *β_2_*.influence inequality when sharing does occur. [↑](#footnote-ref-1)
2. We fix the value of *γ* as it is not a major interest in the model. *γ*=3 is a reasonable value to reflect the percentage of people choosing to share incomes each round in the experiment reported below. [↑](#footnote-ref-2)
3. One example is that actors are extremely altruistic so that the rich give a large proportion of their incomes to the poor, making the poor rich and turning themselves poor. The rich who just turned from poor, motivated by the same altruism, would donate a lot to the poor. Under the circumstance, the rich and the poor constantly exchange their economic status and the game would proceed endlessly. [↑](#footnote-ref-3)
