# Supplementary material for: Good Samaritans in Networks: An Experiment on How Networks Influence Egalitarian Sharing and the Evolution of Inequality
Source: PLoS One. 2015 Jun 10;10(6):e0128777. doi: 10.1371/journal.pone.0128777 (PMC4465669; doi:10.1371/journal.pone.0128777)
Supplement: S3 File — (DOCX) [file pone.0128777.s012.docx]

**S3. Experiment Instruction**

Welcome to the experiment!

Please take out the instruction sheet in the envelope provided.

We will begin with outlining some rules that you must follow during the experiment.

1. Focus on the experiment and do not open any extra Internet browser windows.

2. Do not speak to other participants.

3. If you run into any problems, please raise your hand.

So, you will undergo five different experiments, in which you will use a computer to interact with others in this room. In each experiment, you will be given a certain number of tokens. These tokens are redeemable for real money. We will randomly select one of the five experiments and pay you according to the number of tokens you have at the end of the selected experiment.


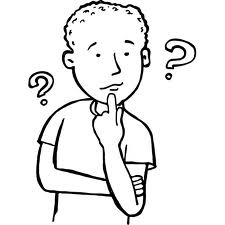


In the experiment, you will first decide whether or not you would like to share your tokens with other participants. Next, you will decide whom you would like to share them with.

Each experiment may last a few rounds. In each round, tokens will be distributed among the participants. You will have a sufficient amount of time to make your transactions. In each round, some participants may choose to share or to no longer share their tokens. As long as there is at least one person allocating his or her tokens, the experiment will continue and progress to the following round. The experiment ends when all transactions have ceased.

You will find five bookmarks, titled “Experiment 1”, “Experiment 2”, “Experiment 3”, “Experiment 4” and “Experiment 5”, located on the top-left toolbar menu of your Mozilla Firefox browser.


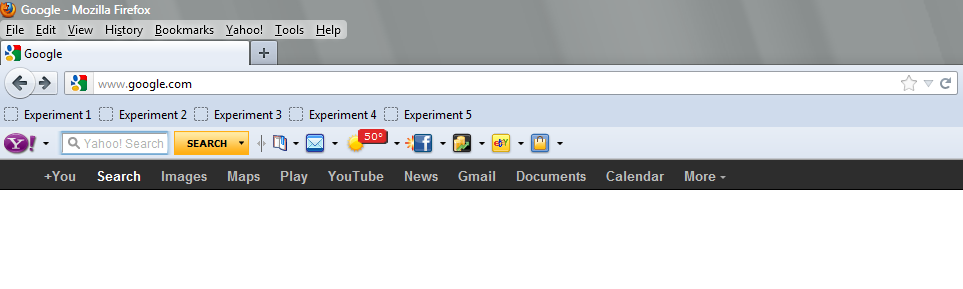
Once instructed to begin an experiment, please click the appropriate bookmark. You will need a Google account and password to log in, which will be provided to you on a slip of paper in your envelope.

Your participation is completely voluntary. If you need to leave please raise you hand and you will be allowed to leave. Once you leave the room, you will not be allowed back inside. If you choose to leave during the experiment, you will receive the 7-dollar show-up payment, but not any of the money earned throughout the experiment.

Are there any questions?

Now please click the bookmark “Experiment 1” to start the first experiment.
