# Supplementary material for: Good Samaritans in Networks: An Experiment on How Networks Influence Egalitarian Sharing and the Evolution of Inequality
Source: PLoS One. 2015 Jun 10;10(6):e0128777. doi: 10.1371/journal.pone.0128777 (PMC4465669; doi:10.1371/journal.pone.0128777)
Supplement: S4 File — (DOCX) [file pone.0128777.s013.docx]

**S4. Statistical Analysis of the Experiment Results**

* All of the analyses were conducted in the statistical program, ***R***.

**S4.1 Comparing the initial- and the end-round inequality levels**

We computed the inequality level (the Gini coefficients) of the income distribution of the initial and the end round in each session. Each session thus contains two inequality values, one for the initial and one for the end round. Using session as the unit of analysis, we employed the Wilcoxon Signed Rank test to check whether the end-round Gini values are significantly different from the initial-round ones for each network treatment.

**S4.2 The Hurdle regression on the probability and the magnitude of giving**

The unit of analysis in the regression is an actor’s giving in a particular round of a particular network treatment. The values of the independent variables, except for the fixed nodal degree (*K*), are pertaining to the round in question.

The hurdle regression was operated in ***R*** by the “pscl” package (Jackman, 2014). Clustering the standard errors of the regression coefficients within individual subjects was done using the package developed by Arai (2009).

**S4.3 Fitting the Beta distribution**

In each round of a treatment, if an actor donated, we calculated the ranking of each of his neighbors’ current income level in the actor’s neighborhood and the proportion of giving they received from the actor. With the two quantities, we can fit them to the Beta distribution to search for the values of two parameters (beta 1 and beta 2) that can best describe the data. We use the package “fitdistrplus” (Delignette-Muller et al., 2014) to operate the fitting of the Beta distribution.

**References**

Arai, M. 2009. Cluster-robust standard errors using R. *URL: http://people.su.se/ma/clustering. pdf*.

Chiang, Y-S., 2013. Cooperation could evolve in complex networks when activated conditionally on network characteristics. Journal of Artificial Society and Social Simulation 16, 6.

Delignette-Muller, M. L., Pouillot, R., Denis J.-B., & Dutang, C. 2014. fitdistrplus: Help to Fit of a Parametric Distribution to Non-Censored or Censored Data.

Erkal, N., Gangadharan, L., Nikiforakis, N., 2010. Relative earnings and giving in a real-effort experiment. American Economic Review 101, 3330-3348.

Gurmu, S., 1998. Generalized hurdle count data regression models. Economics Letters 58, 263-268.

Handcock M. S., Hunter, D.R., Butts, C.T., Goodreau, S.M., Morris, M. 2008. Statnet: Software tools for the representation, visualization, analysis and simulation of network data. Journal of Statistical Software 24, 1548.

Jackman, S. 2014. pscl: Classes and Methods for R Developed in the Political Science Computational Laboratory, Stanford University. Department of Political Science, Stanford University. Stanford, California. R package version 1.4.6. URL http://pscl.stanford.edu/

Morris, M., Handcock, M.S., Hunter, D.R., 2008. Specification of exponential-family random graph models. Journal of Statistical Software 24, 1548.

Robins, G., Pattison, P., Kalish, Y., Lusher, D., 2007. An introduction to exponential random graph (p*) models for social networks. Social Networks 29, 173-191.
